# Nutritional Discrepancies Among Inpatients and Outpatients Diagnosed with Hypertension

**DOI:** 10.3390/healthcare12212119

**Published:** 2024-10-24

**Authors:** Andrzej Wasilewski, Piotr Marczyński, Sylwiusz Kontek, Franciszek Jabłoński, Adrian Kasprzak, Eliza Wasilewska, Aureliusz Andrzej Kosendiak

**Affiliations:** 1 Student Scientific Association, Department of Physical Education and Sport, Wroclaw Medical University, 51-601 Wroclaw, Poland; piotr.marczynski@student.umw.edu.pl (P.M.); sylwiusz.kontek@student.umw.edu.pl (S.K.); adrian.kasprzak@student.umw.edu.pl (A.K.); 2 Faculty of Medicine, Wroclaw Medical University, 50-345 Wroclaw, Poland; franciszek.jablonski@student.umw.edu.pl; 3 Department of Pulmonology and Allergology, Faculty of Medicine, Medical University of Gdansk, 80-210 Gdansk, Poland; ewasilewska@gumed.edu.pl; 4 University of Lower Silesia, Strzegomska 55, 53-611 Wroclaw, Poland; aureliusz.kosendiak@dsw.edu.pl

**Keywords:** cross-centre study, hypertension, nutritional knowledge, BMI, diet

## Abstract

**Objectives:** Arterial hypertension (AH) is one of the most common disorders affecting the human population. The diet of patients with AH can influence the course of the disease and prognosis. The aim of this study was to investigate the differences in nutrition in hospitalised and non-hospitalised hypertensive patients, compared to control groups of non-hypertensive patients from the same medical centres. **Methods:** Patients from nine centres—six hospitals and three ambulatory care centres—were surveyed. The Questionnaire for the Assessment of Dietary Habits, Lifestyle, and Nutrition Knowledge (KomPAN) was administered by interviewers. **Results:** Complete results were obtained from 172 hospitalised and 63 non-hospitalised patients. A significantly higher mean body mass index was found for the hypertensive patients (*p* < 0.001), and a higher unhealthy diet index score was also shown for the hypertensive patients (*p* = 0.003). Over and above this, a lower mean health-promoting diet index score was found in the hospitalised group (who were on a hospital diet) for the hypertensive patients (*p* = 0.018). **Summary:** The study highlights a strong positive correlation between body mass index (BMI) and arterial hypertension (AH), with patients exhibiting higher BMI levels compared to a control group. A BMI of over 25 significantly increases the likelihood of developing AH, and obesity is associated with a higher risk in both men and women. **Conclusions:** The study indicates that a hospital diet may not be suitable for people with AH. Further research should be conducted to obtain reliable results. **Clinical implications:** The study showed which factors should be considered when composing a diet for people with hypertension, the relevance of which was demonstrated in the discussion. The study shows that the problem that clinicians have been struggling with for years is still present and inadequately remedied.

## 1. Introduction

Arterial hypertension (AH) is one of the most common disorders affecting the human population. According to the WHO’s statistics for 2023, an estimated 1.28 billion adults aged 30–79 years suffer from hypertension, with the majority (two-thirds) living in low- and middle-income countries [1]. The trend toward an increase in the incidence of hypertension increases with age. Studies estimate that approximately 90 percent of men and women who are non-hypertensive at 55 or 65 years will develop hypertension by the age of 80–85 [2]. A study conducted in India also suggests a greater increase in the incidence of hypertension in highly urbanized areas (rural areas also see an increase in incidence, but to a lesser extent) [3]. According to the WHO’s statistics, the global trend of hypertension incidence is decreasing, with a simultaneous decrease in the incidence of hypertension in the Americas, Europe and Western Pacific regions, and an increase in the incidence in Africa [2]. Furthermore, it is believed that 46% of adults with hypertension are unaware of their disease [1]. Hypertension significantly increases the risk of cardiovascular events [4]. On the premise of observational considerations, each 10 mmHg increment in SBP is related to a 45% higher hazard of an ischaemic heart infection and almost a 65% higher hazard of ischaemic or haemorrhagic stroke in those aged 55–64. The relative hazard is contrarily related to age [5]. It also contributes to the development of other diseases such as diabetes, atrial fibrillation, and dementia [6]. The definition and categories of hypertension have evolved over the years, but the consensus is that persistent BP values of 140/90 mm Hg or more should be treated, usually using a therapeutic target of 130/80 mm Hg or less [7]. However, depending on age, treatment recommendations may vary. According to the recommendations of the Polish Society of Hypertension (PTNT) in 2019, they recommend starting the therapy of hypertension from a value equal to 160/90 mmHg or above. Therapeutic goals may also differ depending on the diagnosis and age [8]. Various therapies are indicated for the treatment of hypertension, including weight loss in obese patients, sodium reduction, smoking cessation, avoidance of excessive caffeine, and dietary modification such as low saturated fat intake [9]. The first-line pharmacological treatment is ACE inhibitors. Angiotensin II receptor blockers, diuretics, and calcium channel blockers are also used in therapy [10]. There are many obstacles that reduce the effectiveness of hypertension therapy. They are multi-dimensional, resulting from the patient’s non-compliance with recommendations, from the improper explanation of the nature of the disease by the doctor to the patient, and from irregularities in the health system, such as the high prices of drugs [11]. Risk factors for the development of the disease include non-modifiable factors such as gender, age, and genetic factors, as well as numerous modifiable factors including poor diet, smoking, and being overweight [12,13]. The pathophysiology of the disease is complex. The mosaic theory proposed by Page suggest various factors leading to the development of hypertension such as genetics, hemodynamics, environmental, neutral, humoral, adaptive, anatomical, humoral and endocrine [14]. The endocrine component focuses on RAAS, which may contribute to hypertension development by some variants like high-profile renin hypertension, which occurs more often in the young male group. Discussed disorder may also result from hyperactivity of the sympathetic nervous system [15]. Additionally, it is said about the influence of the gut microflora on the development of hypertension. Its disruption is closely linked to the occurrence and development of hypertension [16]. It is also important for patients with AH to be aware of their own disease and the risk factors associated with it, which should allow for better control of the disease [17]. The cornerstone of hypertension prevention and control is appropriate lifestyle modification. In this respect, lack of knowledge and poor attitudes towards lifestyle modification are major obstacles [9]. The aim of this study was to investigate the correlation of modifiable factors—BMI, hospital and outpatient diet and stimulants—with the incidence and severity of hypertension. Outpatient and inpatient populations were considered. Furthermore, the influence of patient knowledge on the course and control of the disease was taken into account.

## 2. Materials and Methods

### 2.1. Study Design

The study was conducted by interviewers conducting interviews with patients in internal medicine wards of Polish hospitals from June to September 2022. In accordance with the Helsinki Declaration, approval was obtained from the bioethics committee at the Wroclaw Medical University No. KB 595/2022. Hospitals and centres were selected to reflect a cross-section of facilities where hypertensive patients are treated. Facilities were selected so that approximately 50% were outpatient clinics, 20% were teaching hospitals, and 30% were district hospitals.

**Figure 1:**
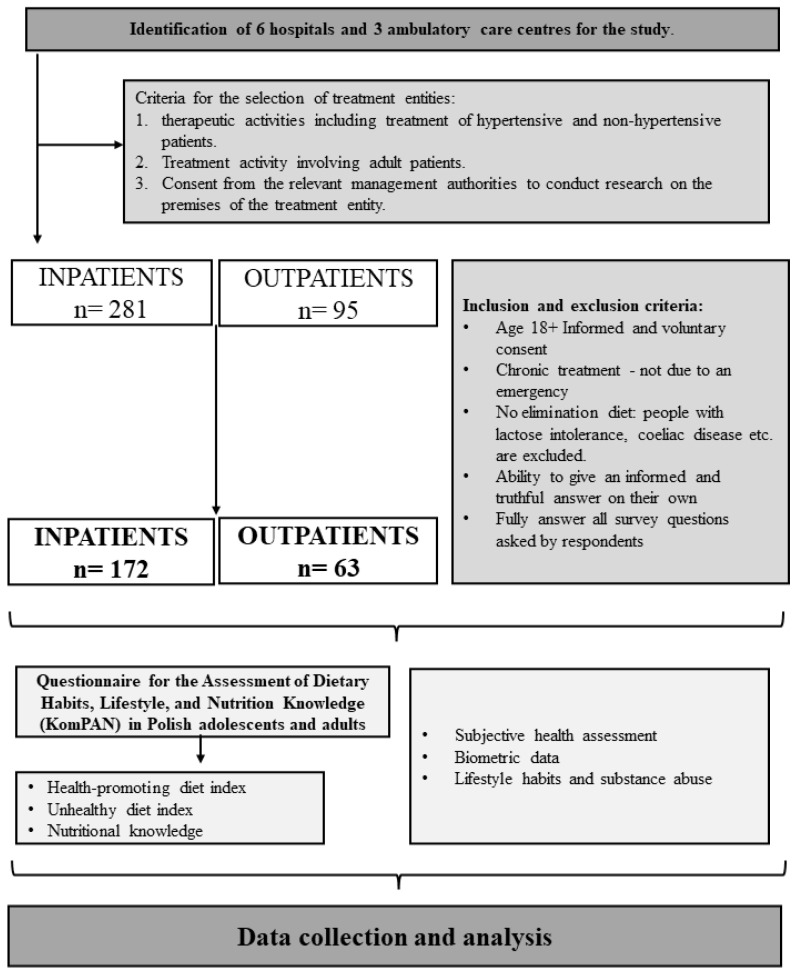
Study design.

The study consisted of a voluntary interview with an interviewer who completed the questionnaire. Interviewers did not collect any personal data, making the study completely anonymous. The interviewers completed the responses via Google Docs. The figure below shows the full inclusion and exclusion criteria for the study. The interviewers surveyed patients who voluntarily agreed to answer their questions. All patients were informed of the voluntariness and anonymity of their responses. All respondents gave their voluntary and informed consent to participate in the study and were informed that they could opt out at any time. The survey consisted of two parts: the Assessment of Dietary Habits, Lifestyle, and Nutrition Knowledge (KomPAN) questionnaire and self-reported questions. A detailed survey, including inclusion and exclusion criteria, is presented in Figure 1.

### 2.2. Sociodemographic Characteristics

Self-reported questions included age, sex, weight, height, marital status, place of residence, ward where the patient is treated, number of previous hospitalisations, and diagnosed diseases. The patient’s subjective health status was also asked; this question could be answered with one of five responses: very bad, bad, neutral, good, and very good. A follow-up question was also asked about diet and whether the patient smokes cigarettes.

### 2.3. Knowledge and Dietary Habits

Nutritional knowledge was examined using the Questionnaire for the Assessment of Dietary Habits, Lifestyle, and Nutrition Knowledge (KomPAN) in Polish adolescents and adults, which is a reproducible validated tool [18]. The questionnaire includes 25 questions regarding nutritional knowledge to which possible answers were true, false and hard to say. For each correct answer, the respondent was assigned 1 point. Insufficient nutritional knowledge was assigned to those who scored 0 to 8 points. Sufficient knowledge for those who scored 9 to 16 points. Those who scored 17 to 25 points were assigned a good level of nutritional knowledge. Eating habits were represented by the Pro Healthy Diet Index (pHDI-10), which included the frequency of consumption of 10 food groups in this category. The Non-Healthy Diet Index (nHDI-14) included the frequency of consumption of 14 unhealthy food groups. A low value of nHDI-14 and pHDI-10 was assigned to respondents with indices of 0 to 33, moderate in the range of 34 to 66, and high in the range of 67 to 100. The pHDI and nHDI were calculated according to the algorithm attached to the questionnaire.

### 2.4. Statistical Analysis

The collected data were processed using Microsoft Excel and Statistica 13.3 (Wroclaw Medical University’s license, StatSoft Polska Sp. z.o.o., Wroclaw, Poland). Body mass index (BMI) was calculated and analyzed based on guidelines from the World Health Organization. Mean, SD, and percentages were calculated to represent the data. Normality of distribution was tested using the Shapiro–Wilk test. Because non-parametric data were obtained, relationships between two independent variables were examined with the Mann–Whitney U test and between several variables with the Kruskal–Wallis test. A *x*^2^ test was used to examine unmeasured characteristics. To make the results presented transparent, percentages were rounded to decimal places, mean and SD values to hundredths, and *p*-values to thousandths.

## 3. Results

### 3.1. Demographic Data

A total of 256 responses were collected, of which, 21 were rejected due to incomplete data or failure to pass the control questions. A total of 143 women and 92 men took part in the study, divided into a study group of people with hypertension and a control group of people without hypertension, attending the same outpatient care centres or the same hospital wards. The groups also responded homogeneously in terms of a subjective assessment of their health status, where in both cases, more than half of the respondents rated their health as good and none rated it as very bad (Table 1 and Table 2).

### 3.2. Comparison of Inpatient and Outpatient Diets

The primary aim of the study was to investigate the relationship between hospitalisation and the health-promoting diet index (pHDI), unhealthy diet index (nHDI), body mass index (BMI) and nutritional knowledge. The relationships obtained are shown in Table 3. In both the study and control group of hospitalised people, vegetables were the most frequently consumed healthy product, while fish was the least frequently consumed. In both groups, the most frequently consumed unhealthy product was white bread, while the least frequently consumed unhealthy product for those with hypertension was energy drinks, and for those without hypertension was lard and animal fats.

### 3.3. Other Results

Correlation coefficients were determined for BMI and the individual factors studied. Statistically significant correlations were obtained for factors such as nutritional knowledge (−0.335), pHDI (−0.240), nHDI (0.299). Over and above this, a positive correlation of pHDI with nutritional knowledge (0.484) and a negative correlation of nHDI with nutritional knowledge (−0.413) were found. In addition, significant differences were found in the factors studied between males and females, as shown in Table 4.

## 4. Discussion

The study shows a well-known, positive correlation between body mass index and arterial hypertension (AH)—patients with AH had a higher BMI (29.5 ± 12.2) in comparison to the control group (25.1 ± 4.7) (*p*-value < 0.001). Research by Firoz et al. indicated a definite relationship between BMI and hypertension [19], as did a study by Chen and Cheng [20]. Having a BMI of over 25 increases the probability of AH by 3.05 times [21]. According to the data of H. Speer et al., obese men were 2.3 times more likely to suffer from AH, (females 1.7×) [22]. Setting aside the high BMI issue, overweight and obese people are often less physically active [23], which also progresses AH [24]. Regardless of BMI, waist circumference is also an indicator associated with AH [25]. Aging is also a risk factor of AH and our study group was statistically older (55.3 ± 14.5) than the control group (44.2 ± 16.9). Our observations coincide with the studies that confirm an age relationship with AH [20]. Although the percentage of smokers was slightly higher in the group without AH—24.8% against 17% of patients with AH—it was not statistically significant in our study, probably due to the number of responses (256). There is no vagueness about the strong harmful hypertensive effect of smoking cigarettes [26].

The study revealed that patients with AH have a higher score on the unhealthy diet index (nHDI) than the health-promoting diet index (pHDI). There is a correlation between AH and the amount of unhealthy food in patients’ diets. For instance, patients struggling with AH pay less attention to the amount of salt in their diet [27]. Though patients received the necessary information about what food should they avoid, two-thirds of them did not follow any recommended diet [28]. Moreover, the diet quality of patients with AH deteriorated by the time of DASH introduction, probably due to social trends [29]. The most difficult issues that patients struggle with when taking care of their dietary choices are social and environmental barriers, costs, adherence to recommendations, emotional and psychological factors and the personal taste preferences of the patient and their family [30]. According to the research of Barbosa et al., processed foods increase the risk of AH in adult and elderly populations [31]. The diet quality score of Australian men correlates with obesity-associated hypertension [32]. Higher consumption of ultra-processed food (UPF) increases the risk of developing AH by 23% compared to a group with low UPF intake [33]. The health threat is even more significant—a UPF-rich diet is associated with metabolic dangers such as obesity and diabetes [34].

The study depicted a subjective health assessment between a study group of patients with hypertension and a group of patients without hypertension. As was shown in the results, the main difference is between neutral and very good subjective perspectives on health topics with a shift in the number of patients with a neutral point of view about their health from very good in the group of patients treated for hypertension. Although it is impossible to eliminate other coexisting diseases, or their absence, which could influence the health assessment, it is surprising that the proportion of health assessments among the patients in both the compared groups is similar. Although hypertension is a serious disease and can lead to numerous complications [35], patients are probably not adequately informed about the health consequences or have never had contact with a person with complications resulting from hypertension [36]. This might lead to questioning the quality of medical education of patients regarding hypertension. Another reason for patients’ high subjective assessment of their health is their psychological tendency to consider their health as positive as it could be; moreover, as has been proven, middle-aged people and the elderly describe their health in comparison to their peers [37]. Other studies also indicate that the subjective assessment of a patient’s health in relation to their physical health and fitness decreases significantly in age groups with an average age over 60 [38], while in the presented study, the age of patients is on average lower, which may also result in an overestimation of health assessments.

In both groups of outpatients and hospitalized patients, the level of nutritional knowledge was clearly higher in people who did not suffer from hypertension. Current research shows a very strong correlation between poor nutritional knowledge and an incorrect diet with both excess body weight and hypertension. Patients with poor nutritional knowledge have a greater tendency to develop hypertension in middle age [39]. Poor nutritional knowledge may lead to making incorrect dietary decisions, especially those that are highly correlated with hypertension, for example, high salt intake [40]. However, this study did not separate eating habits and nutritional knowledge into those that carry an increased risk of hypertension, or those that do not always correlate with hypertension but may negatively impact the patient’s health in other ways. This issue is an aspect to be developed in future research. Current nutritional knowledge can effectively help in the treatment and control of hypertension by limiting the consumption of alcohol and sodium, increasing the intake of magnesium and potassium [41] and introducing changes in eating habits that help reduce body weight, which is necessary in many cases of hypertension [42]; however, it can be suspected that, given the results of this study, many patients may not be aware of the basic health recommendations for people with hypertension, which is confirmed by studies from other regions of the world [39]. Not only nutritional errors resulting from ignorance and excessive intake of food and the resulting large amount of salt can lead to hypertension, but malnutrition of the patient with essential ingredients such as magnesium and calcium also highly correlates with the presence of hypertension [43]. Therefore, it is important to emphasize not only a healthy diet but also a well-balanced diet tailored to the patient’s needs [44].

Regardless of the treatment, inpatients or outpatients with AH had a higher BMI than the control group. The mean BMI of inpatients in general was slightly higher than outpatients. People with a high body weight develop the most significant increase in blood pressure while aging [45]. According to the study of Kuźma et. al., patients with AH and overweight or obese were significantly younger than patients with a regular BMI [46]. Research by M E Díaz showed that a BMI > 25 kg/m^2^ produced a total of 74.4% of patients with AH [47]. The study of F. Javed demonstrated that AH patients had a higher mean BMI, and a higher proportion of overweight and obese categories in comparison to normotensive patients [48].

The nutrition of ambulatory patients with hypertension was significantly worse in comparison to the patients without hypertension (nHDI). However, detailed data are missing, therefore, further study is needed. The data suggest that during hospitalisation, the nutrition of patients without AH was barely worse than their regular diet, in contrast, patients with hypertension clearly reduced their intake of unhealthy products. According to the study of J. Wyka, the nutrition in most of the examined hospitals is unacceptable in terms of quality and quantity and was unpalatable in the opinion of the patients [49]. The Polish State Sanitary Inspection pointed out that in more than 67% of the controlled entities, no improvements were detected. The Supreme Audit Office insisted that, when comparing reports from years 2008 to 2016, no improvements happened [50].

Another potentially important aspect that can be observed in the results is the visible disproportion between hospitalized and outpatients. Although their hospital stay cannot be clearly linked to the presence of hypertension or complications resulting from this condition, a holistic approach to patient treatment should be considered [51], and an attempt to take steps to increase the nutritional awareness of patients under hospital care. Other research shows that the percentage of patients suffering from hypertension whose knowledge about non-pharmacological methods of hypertension therapy is high [52]. Attempts to control hypertension therapy using non-pharmacological methods in current clinical knowledge show high effectiveness in reducing blood pressure, waist circumference and general well-being [53]. Increasing the level of nutritional knowledge among the population in Poland could influence the percentage of patients with hypertension and improve the health service and the economic situation of society. However, the limitation of this solution is the percentage of patients who follow the recommendations they have already learned. Surveys conducted in Poland have shown that more than half of people with uncontrolled hypertension, despite knowing dietary recommendations, do not follow them [54]. Despite this, it is worth seriously considering increasing the emphasis on patient education in the field of nutrition, as meta-analyses show that it helps to significantly reduce the number of patients readmitted to the hospital, which translates into additional positive economic benefits [55]. Patients often obtain false information regarding proper health prevention from unreliable sources [56], so it is worth addressing the problem of the lack of an appropriate number of qualified employees to manage patients through non-pharmacological therapy who would cooperate with medical units.

As shown in the study, nutritional knowledge positively correlates with the intake of more healthy products and negatively correlates with the amount of unhealthy products. Other studies indicate a clear, although small, correlation between the state of nutritional knowledge and a negative dietary pattern [57], which was confirmed among the examined population of Polish patients. The difference in BMI between the sexes may result from poorer nutritional knowledge and poorer dietary decisions [58], but this difference may also result from differences in body structure and composition [59]. Unhealthy food products are more often chosen by men, while women more often choose healthy food products, which is confirmed by subsequent studies on young people; however, it is indicated that significant differences in higher social groups will not occur [60]. Another aspect that may influence the difference between women and men is the biochemical aspect. Metabolism between the sexes shows some significant differences, so it is important that future studies consider trying to differentiate dietary recommendations depending on gender [61]. Maintaining a healthy weight is key to treating hypertension, and an appropriate diet helps achieve therapeutic goals [62]. Moreover, maintaining a normal BMI also helps reduce mortality in other diseases such as cancer [63] and lung diseases [64].

## 5. Conclusions

The study took place in only nine selected centres due to the fact that it is a pilot; further studies should be carried out with sample size calculation.

The conclusions drawn from the study cannot be deemed definitive due to the limited sample size; however, they provide valuable insights and suggest potential hypotheses and directions for future research. Despite this limitation, the implementation of systemic measures involving all medical facilities should occur as soon as possible. Enhanced nutritional education for patients should be prioritized, and efforts should focus on reducing BMI in hypertensive patients. Additionally, hospital diets should be evaluated with respect to the inclusion of healthy and unhealthy products. More factors that could potentially influence BMI—such as medication and duration of illness—should be included in future studies.

## Figures and Tables

**Table 1:** Demographic data.

	Study Group with Hypertension (n = 106)	Control Group Without Hypertension (n = 129)	*p*-Value
Sex n (%)			-
male	52 (49.1%)	40 (31.0%)	
female	54 (50.9%)	89 (69.0%)	
Age, mean ± SD	55.3 ± 14.5	44.2 ± 16.9	-
BMI, mean ± SD	29.5 ± 12.2	25.1 ± 4.7	<0.001 *
Smokers/No smokers, n (%)	18 (17.0%)/88 (83.0%)	32 (24.8%)/97 (75.2%)	0.054

* *p* < 0.05; BMI—Body Mass Index.

**Table 2:** Diet and subjective health assessment in the study and control group.

	Study Group with Hypertension (n = 106)	Control Group Without Hypertension (n = 129)	*p*-Value
Diet			
Health-promoting diet index (pHDI), mean ± SD	36.1 ± 4.8	37.3 ± 5.4	0.108
Unhealthy diet index (nHDI), mean ± SD	40.1 ± 9.2	38.0 ± 9.2	0.003 *
Subjective health assessment, n (%)			
Very Bad	0 (0)	0 (0)	0.471
Bad	17 (16.0%)	16 (12.4%)	
Neutral	31 (29.2%)	29 (22.5%)	
Good	54 (50.9%)	68 (52.7%)	
Very good	4 (3.9)	16 (12.4%)	

* *p* < 0.05; pHDI—health-promoting diet index; nHDI—unhealthy diet index.

**Table 3:** The relationship between hospitalisation and the health-promoting diet index (pHDI), unhealthy diet index (nHDI), body mass index (BMI) and nutritional knowledge.

	Outpatients			Inpatients			*p*-Value Outpatient vs. Inpatient
	With Hypertension (n = 31)	Without Hypertension (n = 32)	*p*-Value Study vs. Control	With Hypertension (n = 75)	Without Hypertension (n = 97)	*p*-Value Study vs. Control	
pHDI mean ± SD	37.6 ± 4.3	36.9 ± 5.1	0.482	35.5 ± 4.9	37.5 ± 5.5	0.018 *	0.419
nHDI mean ± SD	41.5 ± 9.4	37.1 ± 8.3	0.055	39.5 ± 9.0	38.3 ± 9.5	0.338	0.846
Nutritional knowledge mean ± SD	14.2 ± 4.6	16.8 ± 4.8	0.019 *	13.9 ± 4.8	15.6 ± 5.7	0.016 *	0.364
BMI mean ± SD	28.1 ± 4.1	24.1 ± 4.2	<0.001 *	28.5 ± 4.5	25.4 ± 4.8	<0.001 *	0.417

* *p* < 0.05; BMI—Body Mass Index; pHDI—health-promoting diet index; nHDI—unhealthy diet index.

**Table 4:** Relationship between sex and the healthy diet index (pHDI), unhealthy diet index (nHDI), body mass index (BMI) and nutritional knowledge.

	Male n = 92	Female n = 143	*p*-Value
pHDI mean ± SD	34.2 ± 4.6	38.5 ± 4.6	<0.001 *
nHDI mean ± SD	42.8 ± 9.7	36.5 ± 8.2	<0.001 *
Nutritional knowledge mean ± SD	12.9 ± 5.6	16.5 ± 4.5	<0.001 *
BMI mean ± SD	27.8 ± 3.9	25.8 ± 5.2	<0.001 *

* *p* < 0.05; BMI—Body Mass Index; pHDI—health-promoting diet index; nHDI—unhealthy diet index.

## Data Availability

Data are contained within the article.
